# Inhibition of ADORA3 promotes microglial phagocytosis and alleviates chronic ischemic white matter injury

**DOI:** 10.1111/cns.14742

**Published:** 2024-05-07

**Authors:** Yuhao Xu, Limoran Tang, Chao Zhou, Liang Sun, Yujie Hu, Zhi Zhang, Shengnan Xia, Xinyu Bao, Haiyan Yang, Yun Xu

**Affiliations:** ^1^ Department of Neurology, Nanjing Drum Tower Hospital, Affiliated Hospital of Medical School Nanjing University Nanjing China; ^2^ State Key Laboratory of Pharmaceutical Biotechnology and Institute of Translational Medicine for Brain Critical Diseases Nanjing University Nanjing China; ^3^ Jiangsu Key Laboratory for Molecular Medicine Medical School of Nanjing University Nanjing China; ^4^ Nanjing Neurology Clinical Medical Center Nanjing China

**Keywords:** adenosine A3 receptor, Mertk, microglia, phagocytosis, vascular dementia, white matter injury

## Abstract

**Background:**

Adenosine A3 receptor (ADORA3) belongs to the adenosine receptor families and the role of ADORA3 in vascular dementia (VaD) is largely unexplored. The present study sought to determine the therapeutic role of ADORA3 antagonist in a mouse model of VaD.

**Methods:**

The GSE122063 dataset was selected to screen the differential expression genes and pathways between VaD patients and controls. A mouse model of bilateral carotid artery stenosis (BCAS) was established. The cognitive functions were examined by the novel object recognition test, Y maze test, and fear of conditioning test. The white matter injury (WMI) was examined by 9.4 T MRI, western blot, and immunofluorescence staining. The mechanisms of ADORA3‐regulated phagocytosis by microglia were examined using qPCR, western blot, dual immunofluorescence staining, and flow cytometry.

**Results:**

The expression of ADORA3 was elevated in brain tissues of VaD patients and ADORA3 was indicated as a key gene for VaD in the GSE122063. In BCAS mice, the expression of ADORA3 was predominantly elevated in microglia in the corpus callosum. ADORA3 antagonist promotes microglial phagocytosis to myelin debris by facilitating cAMP/PKA/p‐CREB pathway and thereby ameliorates WMI and cognitive impairment in BCAS mice. The therapeutic effect of ADORA3 antagonist was partially reversed by the inhibition of the cAMP/PKA pathway.

**Conclusions:**

ADORA3 antagonist alleviates chronic ischemic WMI by modulating myelin clearance of microglia, which may be a potential therapeutic target for the treatment of VaD.

## Graphical abstract

ADORA3 antagonist promotes the expression of Mertk and Axl in microglia by facilitating cAMP/PKA/p‐CREB pathway, enhances microglial phagocytosis to myelin debris, and improves white matter injury and cognitive impairment in BCAS mice.

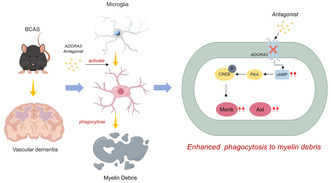

## 1 INTRODUCTION

Vascular dementia (VaD) is the second most common type of dementia after Alzheimer's disease and is associated with a variety of cerebrovascular diseases.^1^, ^2^ Chronic cerebral ischemia is considered to be the main causative factor of VaD.^3^, ^4^ In chronic cerebral ischemia, white matter is destroyed and produce large amounts of myelin debris, which accumulates and promotes increased inflammation and inhibition of axonal regeneration, contributing to the development of cognitive impairment.^5^, ^6^ Therefore, promoting the clearance of myelin debris after chronic ischemia may have a protective effect on the cognitive function of VaD. However, related research and intervention measures are still insufficient.

Microglia are innate immune effector cells with phagocytic and clearance functions, playing a role in clearing debris in the central nervous system during development and disease processes.^7^ After chronic cerebral hypoperfusion, some microglia adopt a protective phenotype upon recognizing damage markers, engulfing myelin debris in the microenvironment, reducing inflammatory reactions, and maintaining microenvironment stability.^8^, ^9^, ^10^ In VaD mice induced by chronic cerebral hypoperfusion, it has been confirmed that promoting the expression of the phagocytic receptor Mertk can enhance microglial phagocytosis of myelin debris, thereby improving WMI and cognitive impairment.^5^ Thus, promoting microglial phagocytosis of myelin debris plays a crucial role in the treatment of VaD.

In the process of searching for key genes regulating phagocytosis of microglia in VaD, we paid attention to adenosine A3 receptor (ADORA3) through the analysis of public databases. ADORA3 is a G‐protein‐coupled receptor that belongs to the adenosine receptor families, which exerts a physiological role by decreasing intracellular cAMP and has been involved in a range of disorders including cardiovascular, neurological, and oncological disorders.^11^, ^12^, ^13^, ^14^ The previous studies revealed that the activation of the cAMP/PKA pathway could promote phagocytosis in microglia.^15^, ^16^, ^17^ Since the activation of ADORA3 inhibits cAMP, whether the inhibition of ADORA3 could protect against VaD by promoting the phagocytosis of microglia has also attracted our attention.

In this study, we discovered ADORA3 as a key gene for VaD and found elevated expression of ADORA3 in the brain of VaD patients in the GSE data. Bilateral common carotid artery stenosis (BCAS) surgery was conducted to mimic a mouse model of VaD from the perspective of chronic ischemic white matter injury (WMI). We observed the effects of ADORA3 antagonist on WMI and cognitive impairment in BCAS mice and expect to provide a new therapeutic strategy to alleviate VaD.

## 2 MATERIALS AND METHODS

### 2.1 Dataset selection and differential gene screening

The dataset GSE122063^18^ was selected from the GEO database (https://www.ncbi.nlm.nih.gov/geo/), which included microarray data from the frontal and temporal lobes of 9 VaD patients and 11 non‐dementia patients. The microarray data underwent analysis using the limma package within the R software, and the mRNA expression differences in the frontal lobe and temporal lobe of the VaD and non‐dementia patients were compared, respectively. *p* < 0.05 and |log2 foldchange>1| were set as the cut‐off values for screening differential genes. Subsequently, the differential genes in the frontal lobe and the differential genes in the temporal lobe between VaD patients and non‐dementia patients were observed, and differential genes with consistent changes in the frontal lobe and temporal lobe were selected for further analysis.

### 2.2 Screening of key genes

The screened differential genes were selected and the protein–protein interaction network diagram was generated using the STRING website (https://string‐db.org/). The network data were then imported into Cytoscape software. Subsequently, in the cytoHubba plugin within Cytoscape software, the clustering coefficient algorithm, which measures the degree of node aggregation based on node triadicity, and the eccentricity algorithm, which assesses the influence of other node proteins on a node based on node centrality, were selected for joint screening of key genes.^19^, ^20^, ^21^

### 2.3 Experimental animal grouping and drug treatment

C57BL/6 male mice (8‐week‐old) were provided by the Animal Model Institute of Nanjing University. The animals were housed at a temperature of 20–25°C, humidity of 45%–60%, and regular alternation of light and darkness (12 h/12 h). Mice were randomly divided into six groups: sham operation group (sham group), Sham+MRS1523 (ADORA3 antagonist) group, BCAS+Vehicle group, BCAS+MRS1523 group, BCAS+H‐89 (cAMP/PKA pathway inhibitor) group, and BCAS+MRS1523+H‐89 (inhibitor of cyclic AMP‐dependent PKA) group. The sham group and BCAS+Vehicle group were all injected intraperitoneally with solvent [DMSO(10%), PEG300(40%), 5% Tween‐80(5%), and saline(45%)] 0.2 mL. The Sham+MRS1523 and BCAS+MRS1523 group was injected intraperitoneally with MRS1523 (MedChemExpress, HY‐1211119, 1 mg/kg). The BCAS+H‐89 group was intraperitoneally injected with H‐89 (MedChemExpress, HY‐15979, 2 mg/kg). The BCAS+MRS1523+H‐89 group was intraperitoneally injected with MRS1523 (1 mg/kg) and H‐89 (MedChemExpress, HY‐15979, 2 mg/kg). The doses of ADORA3 antagonist and H‐89 used in mice were referenced from previous literature.^22^, ^23^ All procedures were approved by the Medical Ethics Committee of Nanjing Drum Tower Hospital (2023AE01011).

### 2.4 Mouse model of bilateral carotid artery stenosis (BCAS)

The mouse model of BCAS was generated in accordance with the methods previously described.^24^, ^25^ Initially, mice were anesthetized by intraperitoneal injection of avertin (2.5%, Sigma). The bilateral common carotid arteries were carefully exposed and then encircled with microcoils (inner diameter 0.18 mm, spacing 0.50 mm, and total length 2.5 mm, purchased from Sawane Spring Co.). Sham‐operated mice underwent all procedures except for the implantation of microcoils.

### 2.5 Laser speckle contrast imaging

The method of laser speckle contrast imaging was employed to assess cerebral blood perfusion in this study. The High‐Resolution Laser Speckle Contrast Imager (LSCI) from PeriCam PSI System, a company based in Sweden, was used. To prepare the mice for imaging, they were initially anesthetized with Avertin, a 2.5% solution from Sigma. Subsequently, the fur on the mice was shaved to expose the skull. A midline cross‐skin incision was made to further expose the skull, which was then cleaned using a cotton applicator soaked in saline solution. To quantify hemispherical perfusion, a region of interest (ROI) was defined between the bregma and lambda, specific anatomical landmarks on the skull. This ROI facilitated the measurement of blood flow from both sides of the brain. The imaging procedure was carried out using the PSI system, with a camera positioned 10 cm above the skull to capture the cerebral blood flow (CBF) perfusion images. The acquired images were analyzed using the customized PIMSoft program (Perimed Inc.). BCAS mice exhibit reduced CBF (Figure S1).

### 2.6 Novel object recognition (NOR) test

The NOR test was conducted following a previously described protocol.^25^ The first part of the experiment involved an adaptation test, where the mice were placed in a box with no objects for a duration of 15 min. This test was repeated for 3 consecutive days. After the adaptation test, the mice entered the training phase. In this phase, they were exposed to two identical objects and given 10 min to explore each object. This training phase was also conducted for 3 consecutive days. Following the completion of the training phase, the mice were given a 1‐h break. After this break, the mice were placed back into the same box used for the adaptation test. However, this time, one of the objects from the training phase was replaced with a new object. Mice with normal cognitive function would spend more time exploring new objects, whereas BCAS mice show reduced exploration time toward new objects. The total exploration time each mouse spent on both objects in the box was recorded and subjected to analysis.

### 2.7 Y‐maze test

The Y‐maze test was conducted following a previously established procedure.^24^ The Y‐maze, constructed from dark polyethylene plastic, comprises three arms labeled A, B, and C. Individual mice were put into the arm junctions and allowed to explore for 8 min freely. Spontaneous alternations were documented when mice consecutively entered all three different arm sequences such as ABC, ACB, BAC, BCA, CBA, and CAB. The percentage of spontaneous alternations was calculated as the number of behavioral spontaneous alternations divided by the total number of arm entries minus 2, multiplied by 100%. Mice with normal cognitive function would demonstrate good spontaneous alternations, whereas BCAS mice would exhibit reduced spontaneous alternations.

### 2.8 Fear of conditioning (FC) test

Fear of conditioning tests were conducted to assess context‐dependent and cue‐dependent memory, following a previously established protocol.^25^ Briefly, mice underwent a 5‐min acclimatization period in a conditioning chamber, during which a 2 Hz pulse tone (80 dB, 3600 Hz) was emitted for 30 s, followed by a transient electric shock (0.8 mA, 1 s) to the foot. Testing took place the day after the aforementioned training. Context‐dependent memory was evaluated by placing the mice in a conditioning chamber with the same background for 5 min, and the proportion of total time spent in freezing behavior was recorded. In the case of cue‐dependent memory, mice were placed in a conditioning chamber with a completely different background. They were allowed 1 min of exploration without stimulation, followed by 4 min of exploration under pulsed tone stimulation. The proportion of time spent exhibiting freezing behavior was then recorded. Mice with normal cognitive function would demonstrate good context‐dependent memory and cue‐dependent memory, whereas BCAS mice would exhibit impaired context‐dependent and cue‐dependent memory.

### 2.9 Magnetic resonance imaging (MRI)

Magnetic resonance imaging was conducted on a 9.4T MRI System (BioSpec 94/20 USR, Bruker). T2‐weighted images were acquired using a 2D RARE sequence with the following parameters: repetition time of 2500 ms, echo time of 33 ms, matrix size of 256 × 256, field of view of 20 mm × 20 mm, and 22 neighboring slices with a slice thickness of 0.7 mm. For diffusion‐weighted images, a spin‐echo planar imaging sequence was employed with b‐values of 0 and 1000 s/mm^2^, along with 30 noncollinear directions, *δ* = 4.1 ms, Δ = 10.3 ms, repetition time of 1500 ms, echo time of 23.27 ms, matrix size of 128 × 128, field of view of 20 mm × 20 mm, and 22 neighboring slices with a slice thickness of 0.7 mm. Images were converted to NIFTI format using MRIcron. Subsequent data processing was facilitated by the FSL tool. This was followed by b0‐image (BET)‐based brain mask extraction and fractional anisotropy (FA) mapping calculations achieved by fitting a diffusion tensor model at each voxel (dtifit). For the extraction of FA values, ITK‐SNAP mapping was applied.

### 2.10 Western blot analysis

Thirty micrograms of proteins were utilized for sodium dodecyl sulfate–polyacrylamide gel electrophoresis. The membranes were subsequently transferred using a semi‐dry method. Following transfer, the membranes were blocked with a 50 g/L skimmed milk solution for 2 h. Primary antibodies were added overnight at 4°C, including ADORA3 (Alomone Labs, AAR‐004, 1:500), PLP1 (Abclonal, A20009, 1:2000), MAG (Abcam, ab89780, 1:2000), Mertk (Abcam, ab95925, 1:1000), Axl (Abcam, ab215205, 1:1000), p‐CREB (Cell Signaling Technology, 9198, 1:1500), CREB (Cell Signaling Technology, 9197, 1:1500), and GAPDH (Proteintech, 60004‐1‐ig, 1:1000). The membranes were washed with trihydroxymethyl aminomethane salt buffer (TBST) before the addition of secondary antibodies. After incubating for 2 h, the membranes were repeatedly washed with TBST. Finally, luminescence was detected in a dark room, and the samples were developed and photographed for preservation. The relative expression levels were calculated using GAPDH as an internal reference. Tissue and cell samples were isolated using RIPA and protease inhibitors.

### 2.11 Quantitative polymerase chain reaction (qPCR)

cDNA was synthesized through reverse transcription. Quantitative PCR was conducted on the ABI StepOne Plus PCR Instrument. The primer sequences for ADORA3 were as follows: forward primer: CCTGGGGAAGTAAGAACGGT, reverse primer: GACCCAGCTCTTGTCAGACT. The expression levels of the target gene were normalized to the endogenous control GAPDH.

### 2.12 Enzyme‐Linked Immunosorbent Assay (ELISA)

ELISA was conducted to examine the levels of cAMP and PKA following the manufacturer's instructions (R&D Systems Inc).

### 2.13 Immunofluorescence staining

The mice were deeply anesthetized using 2.5% avertin (Sigma) and then perfused with ice‐cold 0.1 mol/L phosphate‐buffered saline (PBS), followed by 4% formaldehyde in 0.1 mol/L PBS. Subsequently, the cerebrums were carefully removed from the cranial fossae and immersed in 15% and 25% sucrose solutions at 4°C for 24 h in each concentration. After sucrose immersion, the brains were sliced into 20 μm sections using a rotary microtome (Thermo Fisher, Waltham, USA). Coronal sections were fixed using 4% paraformaldehyde, followed by blocking with BSA and permeabilization with Triton X‐100. The primary antibodies used were as follows: ADORA3 (Alomone Labs, AAR‐004, 1:100), Iba‐1 (Abcam, ab178847, 1:500), GFAP (Proteintech, 16825‐1‐ap, 1:500), Olig2 (MilliporeSigma, AB9610, 1:500), MBP (Abcam, ab7349, 1:1000), Mertk (Abcam, ab95925, 1:200), and dMBP (Merck, AB5864, 1:500). After incubation with the corresponding secondary antibodies for 2 h, sections were visualized using a fluorescence microscope. The mouse brain anatomical diagrams and MBP immunofluorescence staining images were employed to illustrate the precise locations for image capture in the CC (corpus callosum), EC (external capsule), and STR (striatum) regions (Figure S2). Additionally, the primary microglia and oligodendrocytes were fixed in 4% formaldehyde for 15 min and followed a similar protocol. DiD is a far‐infrared cell membrane fluorescent probe with a maximum excitation wavelength of 644 nm and a maximum emission wavelength of 665 nm. It can display red light under the excitation wave.

### 2.14 Luxol fast blue (LFB) staining

To detect the integrity of myelin, the LFB staining was employed. A specific procedure was followed: firstly, a 0.1% solution of Luxol Fast Blue (purchased from Sigma‐Aldrich) was used to stain a series of sections. These stained sections were then sealed and incubated at a temperature of 60°C for a duration of 8 h. After the sealing process, the sections were washed and subsequently immersed in alcohol (95%) for a period of 10 min. To achieve optimal staining, each section was then subjected to two additional steps. Firstly, the section was immersed in a 0.05% lithium carbonate aqueous solution for a duration of 10 s. This step was followed by immersion in a 70% alcohol solution for a period of 20 s. The aforementioned two steps were repeated in each section until both the gray and white matter could be observed clearly under the microscope. Once the staining process was completed, the sections underwent dehydration using a conventional alcohol gradient. The sections were successively exposed to alcohol concentrations of 80%, 95%, 95%, 100%, and 100% for a duration of 2 min each. Following the alcohol gradient, the sections were immersed in xylene I and II for a total of 10 min. The resulting LFB‐stained images were captured with a microscope (Olympus IX73), and the average optical density (AOD) was quantified using ImageJ. The mouse brain anatomical diagrams and LFB staining images were also utilized to demonstrate the precise locations for image capture in the CC, EC, and STR regions (Figure S2).

### 2.15 Primary microglia culture

Primary microglia were purified from 1‐day‐old C57BL/6J mice.^26^ The cerebral cortex was handled with trypsin EDTA for 10 min. Following this digestion process, an equal amount of DMEM medium supplemented with 10% fetal bovine serum (FBS) was used to terminate digestion. The cells were then subjected to centrifugation at 800 rpm for a duration of 10 min at a temperature of 37°C. After carefully aspirating the supernatant, the cells were transferred to 75 cm^2^ culture flasks and incubated for a period of 10–12 days. During this time, the microglia cells were separated by gently shaking the culture flasks. Floating microglia were transplanted on the plate and cultured for about 48 h. Following the collection of microglia, the cells were subjected to various treatments, including myelin debris (0.01 mg/mL) alone, co‐treatment of myelin debris and MRS1523 (0.1 μM), co‐treatment of myelin debris (0.01 mg/mL) and ADORA3 agonist (IB‐MECA, 0.2 μM), and co‐treatment of myelin debris (0.01 mg/mL) with MRS1523 (0.1 μM) and H‐89 (10 μM). The doses of MRS1523, IB‐MECA, and H‐89 used in cells were referenced from previous literature.^27^, ^28^, ^29^

### 2.16 Myelin debris preparation and stimulation

Myelin debris preparation followed established protocols.^30^, ^31^ In summary, mice were humanely euthanized, and brain samples were isolated and cut into approximately 5 × 5 × 5 mm^3^ sections. The solution was transferred to a 0.83 M sucrose solution. After centrifugation, crude myelin debris was then dissolved in a Tris·Cl buffer solution and subjected to homogenization for a period of 30–60 s. After centrifugation twice, the sediment was resuspended in sterile phosphate buffer solution (PBS) and centrifuged at 4°C for 10 min at 22,000 rpm. Finally, myelin debris was resuspended and a final concentration of 100 mg/mL was acquired. The myelin debris was subsequently stained with DiD (a far‐red plasma membrane fluorescent probe) for flow cytometry and immunofluorescence analysis.

### 2.17 Flow cytometry

Following a 1‐h exposure of primary microglia to DiD‐labeled myelin debris (0.01 mg/mL), ADORA3 inhibitor (MRS1523) and/or H‐89 were introduced for an additional 1‐h co‐incubation period. Subsequent to trypsin digestion, cells were harvested, centrifuged, and resuspended in PBS. The ratio of microglia phagocytosing myelin debris (DiD^+^ cells/total cells) and the fluorescence intensity of DiD were analyzed using flow cytometry (BD Biosciences, Carlsbad, CA, USA).

### 2.18 Primary oligodendrocytes culture

Primary oligodendrocytes originate from the proliferation and differentiation of oligodendrocyte precursor cells (OPCs) based on previous experiments.^31^, ^32^ OPCs were obtained from newborn C57BL/6 pups (postnatal 0–2; P0–2). Following the removal of the meninges, cortical tissues were mechanically minced and dissociated. Subsequently, the tissue suspension was filtered through a 70 μm nylon cell strainer and plated on poly‐D‐lysine‐coated culture plates or dishes with Dulbecco's modified Eagle's medium/F12, B27, and penicillin/streptomycin proliferation medium (Gibco, CA, USA), supplemented with 15 ng/mL platelet‐derived growth factor‐AA (GenScript, Beijing, China) and 5 ng/mL basic fibroblast growth factor (GenScript), for 7–10 days before differentiation. Triiodothyronine (T3; R&D Systems, MN, USA) and ciliary neurotrophic factor (CNTF; GenScript) were employed to induce OPC differentiation. Oligodendrocytes were obtained after 7–10 days of differentiation. Additionally, CoCl_2_ (1 μM) was introduced to oligodendrocytes and cultured for 7 days to simulate chronic hypoxia.^33^ MRS1523 (0.1 μM) was also added to oligodendrocytes and cultured together for 7 days for further testing.

### 2.19 Statistical analysis

Statistical analysis was conducted using Prism 8.0 software (GraphPad Software, USA). Data were presented as mean ± standard deviation (Mean ± SD). The normality of the data was assessed using the Kolmogorov–Smirnov test. For comparisons between two groups, Student's *t*‐test was utilized when continuous variables were normally distributed; otherwise, the Mann–Whitney test was employed. In cases involving multiple comparisons, data were analyzed using one‐way analysis of variance (ANOVA) with Bonferroni post hoc test for normally distributed data and Kruskal–Wallis test for non‐normally distributed data. Statistical significance was set at *p* < 0.05.

## 3 RESULTS

### 3.1 ADORA3 is elevated in VaD patient brains

**FIGURE 1:**
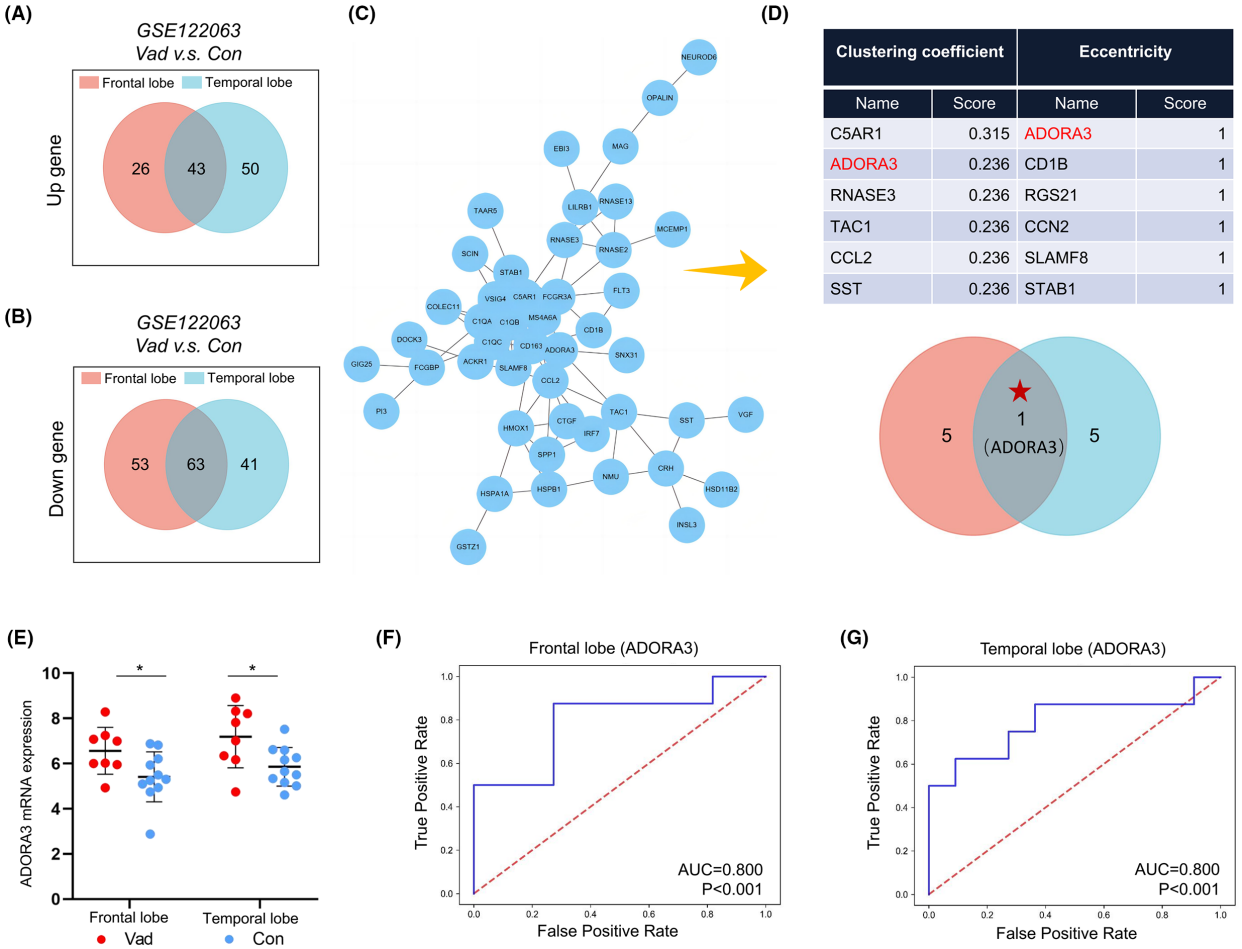
Analysis of ADORA3 in GSE122063. (A, B) Forty‐three genes were simultaneously upregulated and 63 genes were simultaneously downregulated in the frontal and temporal lobes in VaD patients. (C) Protein–protein interaction network diagram of differential genes. (D) The clustering coefficient and eccentricity algorithms screened ADORA3 as a key gene for VaD. (E) ADORA3 expression was significantly elevated in the frontal and temporal lobes of VaD compared to controls. (F, G) ROC curves of frontal and temporal ADORA3 in VaD and controls. *p < 0.05.

Initially, we analyzed the mRNA expression in VaD patient brains in the GSE122063 dataset. Compared with non‐dementia patients, there were 69 upregulated genes and 93 downregulated genes in the frontal lobe, and 116 upregulated genes and 104 downregulated genes in the temporal lobe of VaD patients. There were 106 genes with consistent changes in frontal and temporal lobes in VaD patients, including 43 upregulated genes and 63 downregulated genes (Figure 1A,B). The specific gene names, fold changes, and statistical values are presented in Table S1. Subsequently, we imported these 106 genes into the STRING website to generate a protein–protein interaction network diagram for observing the connections between genes (Figure 1C). The network data were then imported into Cytoscape software, and the results revealed that ADORA3 was identified as a key regulatory gene of VaD in both the clustering coefficient and eccentricity algorithms (Figure 1D). Furthermore, the expression of ADORA3 mRNA was found to be higher in the frontal and temporal lobes of VaD patients compared to non‐dementia patients, and the ROC curve also demonstrated good diagnostic value (Figure 1E–G).

### 3.2 ADORA3 is significantly elevated in microglia after BCAS

**FIGURE 2:**
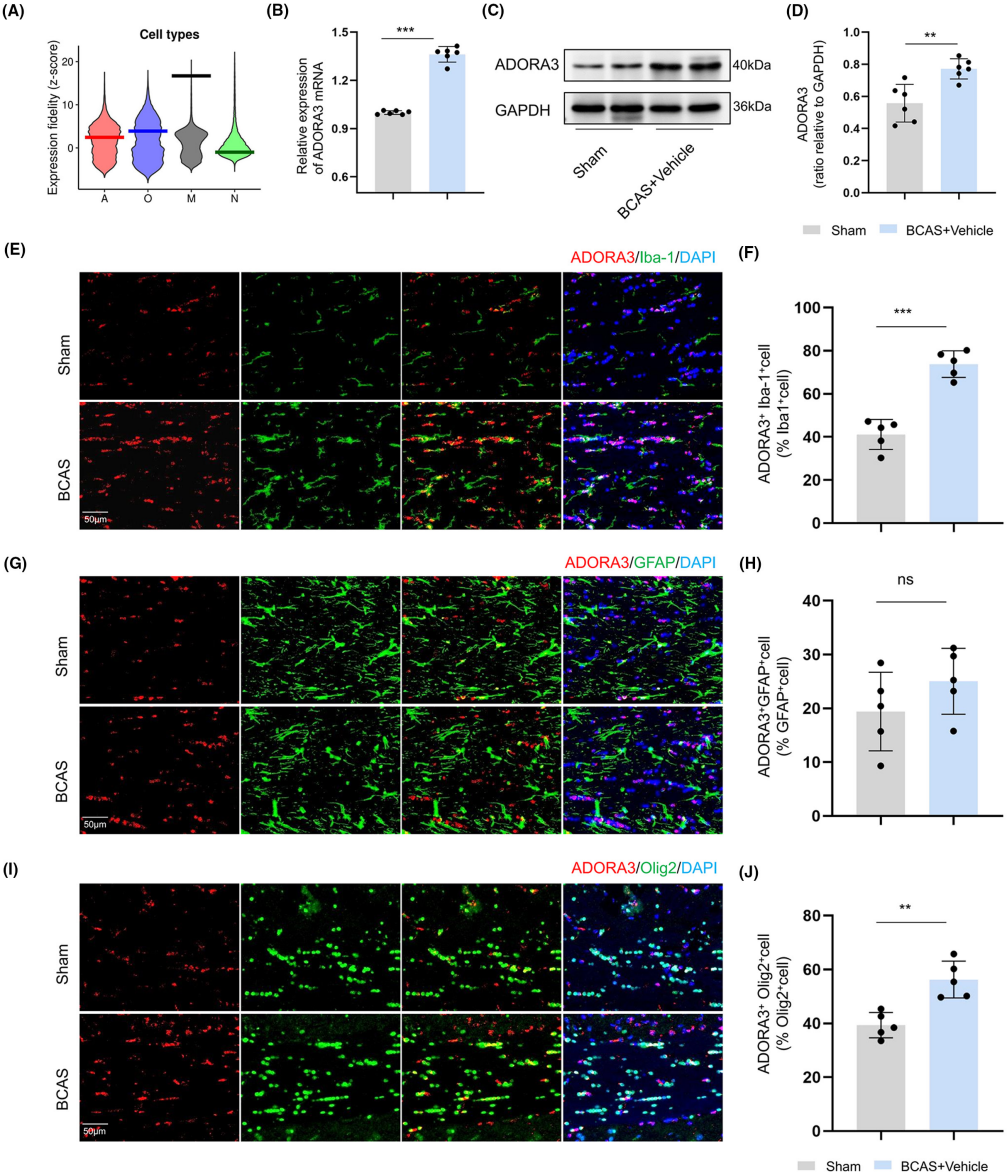
The expression of ADORA3 in the CC of BCAS mice. (A) Expression fidelity of ADORA3 in human brain white matter (http://oldhamlab.ctec.ucsf.edu/; A: astrocyte, O: oligodendrocyte, M: microglia, N: neuron. Expression fidelity is a z‐score that quantifies the extent to which the expression pattern of each gene was correlated with the inferred abundance of the cell type over all queried samples. (B) Elevated ADORA3 mRNA expression in the CC of BCAS mice (*n* = 6). (C, D) Elevated ADORA3 protein expression in the CC of BCAS mice (*n* = 6). (E, F) Immunofluorescence staining and quantitative analysis of ADORA3^+^Iba‐1^+^ cells in the CC (*n* = 5). (G, H) Immunofluorescence staining and quantitative analysis of ADORA3^+^GFAP^+^ cells in the CC (*n* = 5). (I, J) Immunofluorescence staining and quantitative analysis of ADORA3^+^Olig2^+^ cells in the CC (*n* = 5). Scale bar 50 μm. **p < 0.01, ***p < 0.001, “ns” indicates no significance (*p* > 0.05).

In Kevin W's database (http://oldhamlab.ctec.ucsf.edu/), we observed ADORA3 expression in human brain white matter and found that ADORA3 has the highest expression fidelity in microglia in white matter (Figure 2A), which suggests that ADORA3 function may be carried out by microglia. Subsequently, we observed ADORA3 expression in the CC of BCAS mice and found that the expression of ADORA3 mRNA and protein were both elevated after 2 months of BCAS (*p* < 0.01) (Figure 2B–D). Since the white matter is dominated by glial cells, we explored the expression of ADORA3 in oligodendrocytes, astrocytes, and microglia in the CC. The results showed that 2 months after BCAS, the greatest increase in ADORA3 expression was observed in microglia (*p* < 0.001) (Figure 2E,F), followed by oligodendrocytes (*p* < 0.01) (Figure 2G,H), with no significant change in astrocytes (*p >* 0.05) (Figure 2I,J).

### 3.3 ADORA3 antagonist alleviates WMI and cognitive impairment after BCAS

**FIGURE 3:**
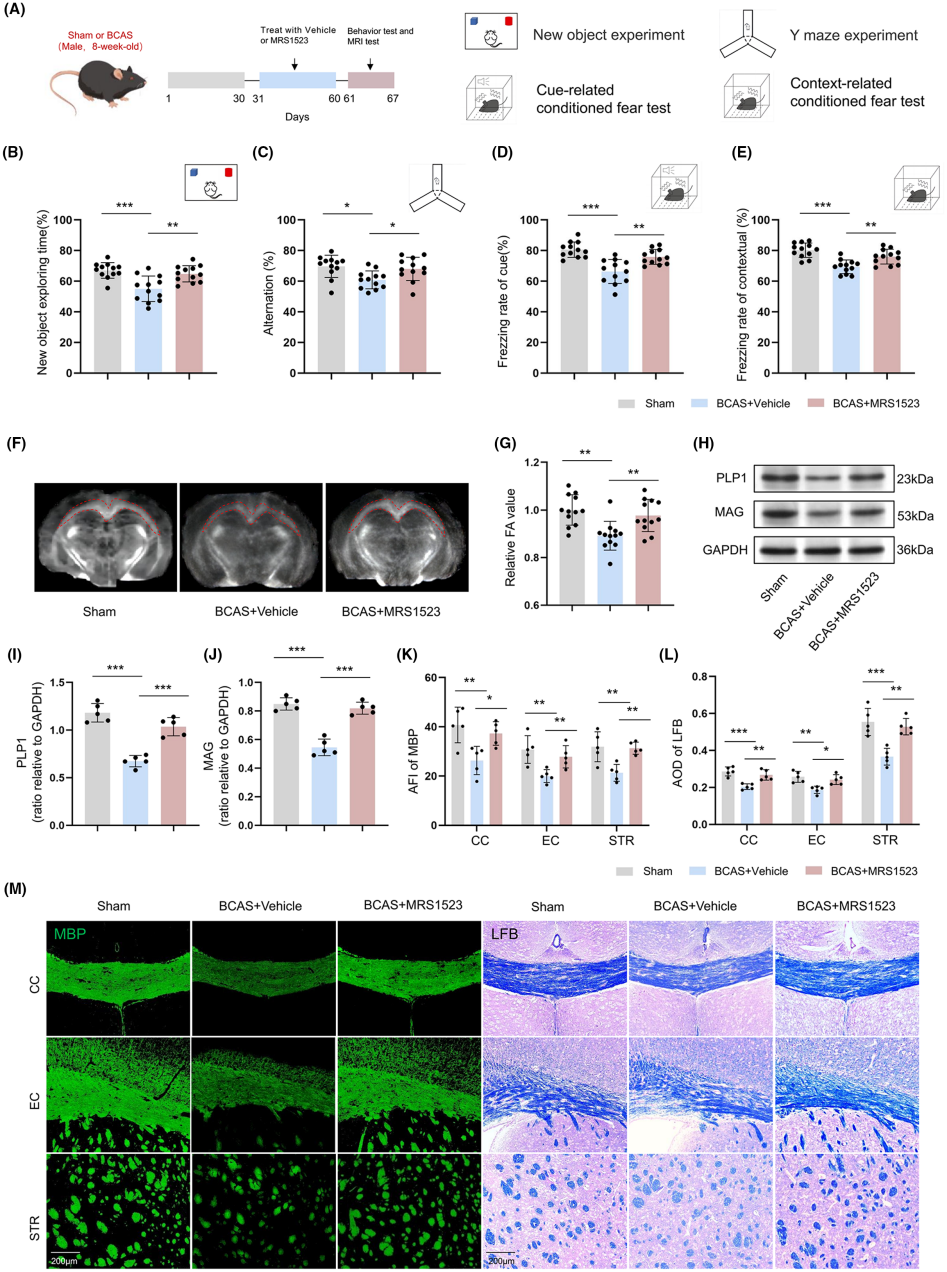
Effects of ADORA3 antagonist on WMI and cognitive impairment after BCAS. (A) Grouping of mice and procedure. (B) NOR test (*n* = 12). (C) Y maze test (*n* = 12). (D) Cue‐related FC test (*n* = 12). (E) Context‐related FC test (*n* = 12). (F, G) Representative FA images and quantitative analysis (*n* = 12). (H–J) Representative western blot images and quantitative analysis of PLP1 and MAG (*n* = 5). (K–M) Immunofluorescence staining and quantitative analysis of MBP and LFB staining in the CC, EC, and STR (*n* = 5). Scale bar 200 μm. *p < 0.05, **p < 0.01, ***p < 0.001.

ADORA3 antagonist (MRS1523) or vehicle was injected into the mice starting from 1 month after BCAS, once daily for 1 month, and behavioral tests were performed subsequently (Figure 3A). Firstly, we observed the effects of MRS1523 on sham mice. After confirming that MRS1523 treatment had no impact on the bodyweight, cognition, or myelin of sham mice (*p* > 0.05; Figure S3), the MRS + Sham group was excluded from further focused observation. Subsequently, we found that vehicle‐treated BCAS mice perform worse in the Y‐maze test, NOR test, and FC test than sham controls, and the MRS1523‐treated BCAS mice perform better in these tests than vehicle‐treated BCAS mice (*p* < 0.05; Figure 3B–E). The MRI DTI results also showed decreased FA values in the CC and EC of vehicle‐treated BCAS mice compared with sham controls, and MRS1523 treatment increased the FA values in the CC after BCAS (*p* < 0.01; Figure 3F,G). Meanwhile, the expression of mature OL myelin phospholipoproteins (PLP1 and MAG) in the CC showed a decrease in vehicle‐treated BCAS mice compared with sham controls, as well as the MBP and LFB intensities in the CC, EC, and STR. However, MRS1523 treatment increased expression of PLP1 and MAG in the CC (*p* < 0.001; Figure 3H–J), and also increased MBP and LFB intensities after BCAS (*p* < 0.05; Figure 3K–M).

### 3.4 ADORA3 antagonist promotes phagocytosis of microglia by upregulating Mertk expression

**FIGURE 4:**
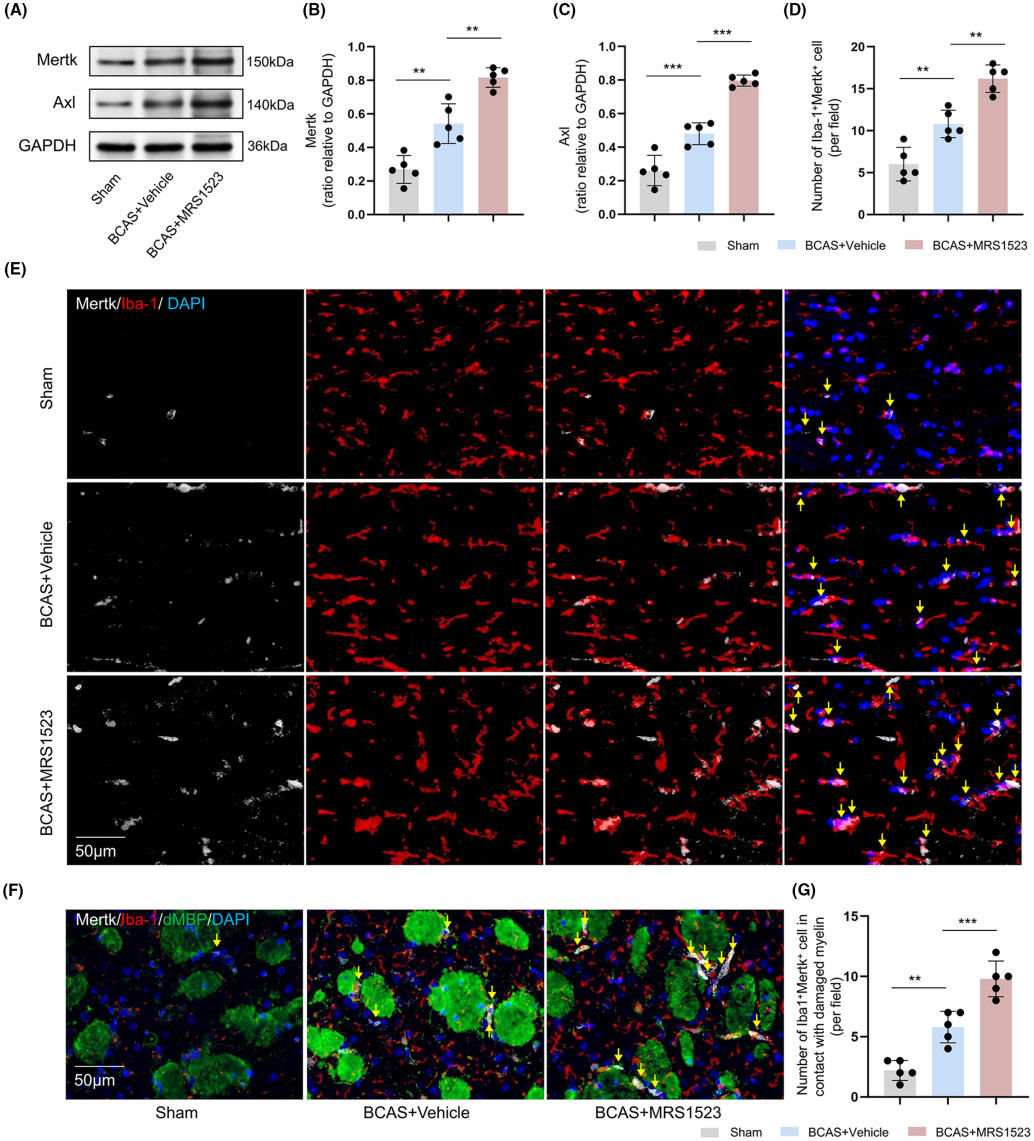
Effects of ADORA3 antagonist on phagocytosis of microglia after BCAS. (A–C) Representative western blot image and quantitative analysis of Mertk and Axl expression in the CC (*n* = 5). (D, E) Immunofluorescence staining and quantitative analysis of Iba‐1^+^Mertk^+^ cells in the CC (*n* = 5). (F, G) Immunofluorescence staining and quantification of Iba‐1^+^Mertk^+^ cells in contact with damaged MBP in the STR (*n* = 5). Scale bar 50 μm, *p < 0.05, **p < 0.01, ***p < 0.001.

We wondered whether ADORA3 antagonist modulated phagocytosis of microglia and examined the expression of Mertk and Axl proteins that respond to cellular phagocytosis. The vehicle‐treated BCAS mice showed higher expression of Mertk and Axl proteins in the CC than sham controls, and the MRS1523 treatment further increased the expression of Mertk and Axl proteins when compared with vehicle‐treated BCAS mice (*p* < 0.01; Figure 4A–C). The number of Iba‐1^+^Mertk^+^ cells in vehicle‐treated BCAS mice was higher than in sham controls, and the MRS1523 treatment further increased the number of Iba‐1^+^Mertk^+^ cells when compared with vehicle‐treated BCAS mice (*p* < 0.01; Figure 4D,E). In addition, in the STR, damaged myelin in the vehicle‐treated BCAS mice was surrounded by Iba‐1^+^Mertk^+^ cells, and the contact between the damaged myelin and Iba‐1^+^Mertk^+^ cells was enhanced after MRS1523 treatment (*p* < 0.01; Figure 4F,G).

### 3.5 ADORA3 antagonist upregulates Mertk expression and microglial phagocytosis by activating cAMP/PKA/p‐CREB pathway

**FIGURE 5:**
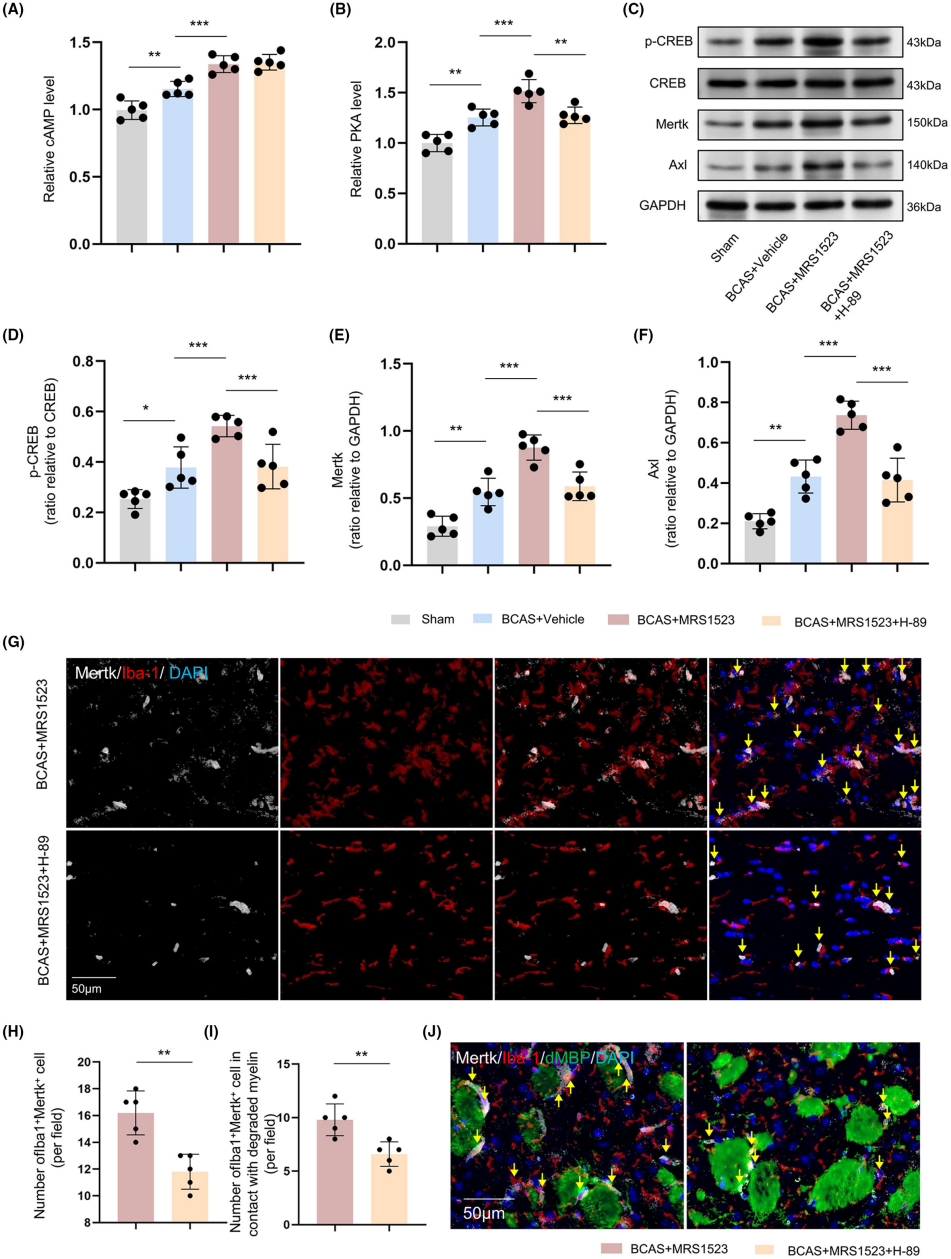
ADORA3 antagonist upregulates Mertk expression and microglial phagocytosis by activation of cAMP/PKA/p‐CREB pathway. (A, B) Relative cAMP and PKA levels in the CC (*n* = 5). (C–F) Representative western blot images and quantitative analysis of CREB, p‐CREB, Mertk, and Axl expression in the CC (*n* = 5). (G, H) Immunofluorescence staining and quantitative analysis of Iba‐1^+^Mertk^+^ cells in the CC (*n* = 5). (I, J) Immunofluorescence staining and quantification of Iba‐1^+^Mertk^+^ cells in contact with damaged MBP in the STR (*n* = 5). Scale bar 50 μm, *p < 0.05, **p < 0.01, ***p < 0.001.

We further analyzed cAMP/PKA/p‐CREB pathway after MRS1523 treatment. The vehicle‐treated BCAS mice showed elevated levels of cAMP and PKA in the CC than sham controls (*p* < 0.01), and MRS1523 treatment further increased the levels of cAMP and PKA when compared with vehicle‐treated BCAS mice (*p* < 0.001; Figure 5A,B). We also examined the expression of p‐CREB, a downstream protein of the cAMP/PKA pathway. The results showed that the p‐CREB protein expression in the vehicle‐treated BCAS mice was higher than sham controls, and MRS1523 treatment increased p‐CREB protein expression when compared with vehicle‐treated BCAS mice (*p* < 0.05; Figure 5C,D), suggesting that there was an increase in the activation of cAMP/PKA/p‐CREB pathway after injected with MRS1523. Additionally, we also evaluated whether cAMP/PKA pathway inhibitor (H‐89) had an effect on the phagocytosis of microglia promoted by MRS1523. The supplement of H‐89 decreased the expression of PKA and p‐CREB, which was boosted by MRS1523 treatment in BCAS mice (*p* < 0.01; Figure 5A–D). Correspondingly, the expression of Mertk and Axl proteins was also reduced after treatment with H‐89 treatment (*p* < 0.01; Figure 5C,E,F). The immunofluorescence results showed that H‐89 treatment decreased the number of Iba‐1^+^Mertk^+^ cells in the CC when compared with MRS1523‐treated BCAS mice (*p* < 0.01; Figure 5G,H), and a weakening of the contact between Iba‐1^+^Mertk^+^ cells and the damaged myelin in the STR was also observed after H‐89 treatment (*p* < 0.01; Figure 5I,J).

### 3.6 H‐89 partially reversed the protective effect of ADORA3 antagonist on WMI and cognitive impairment in BCAS mice

**FIGURE 6:**
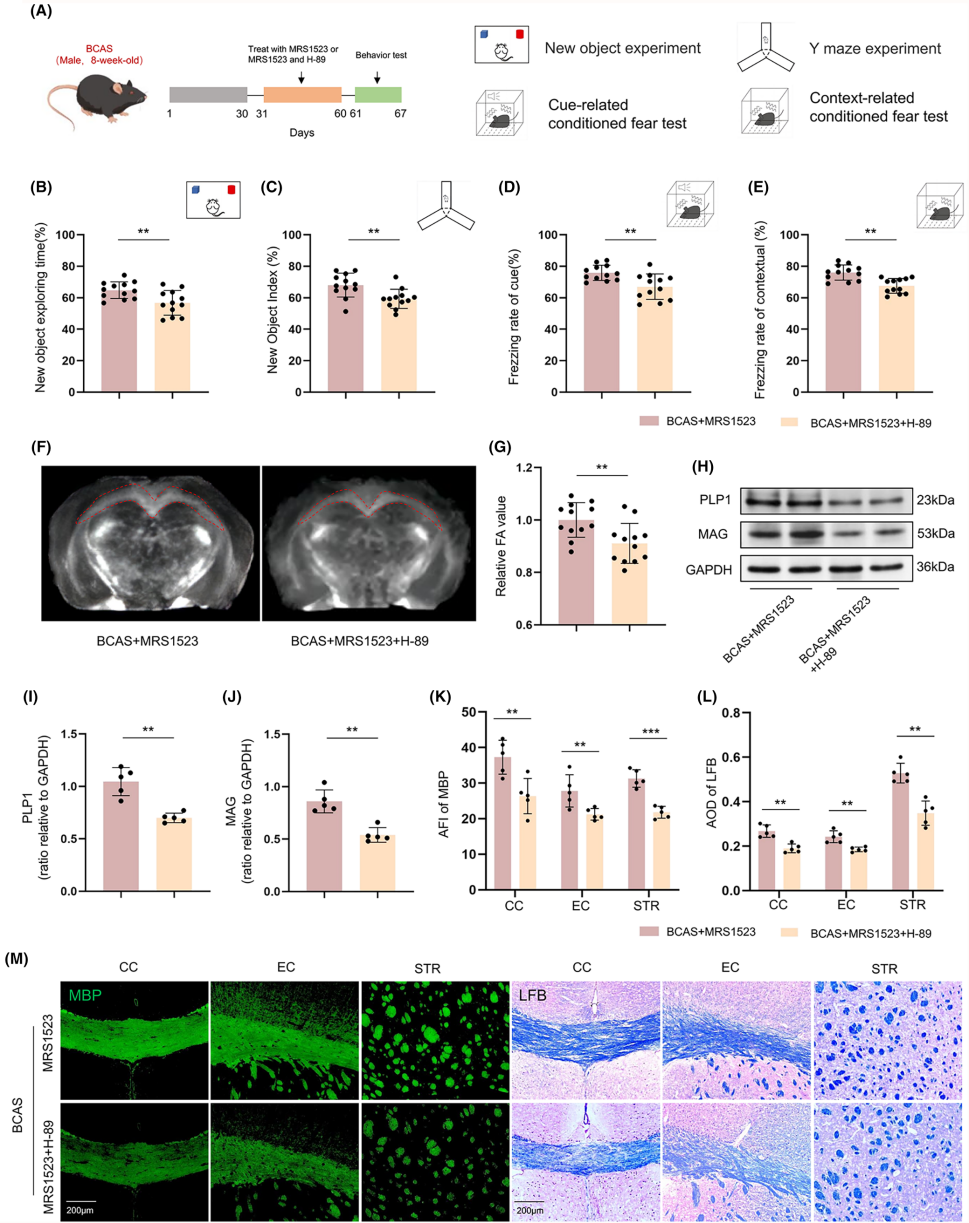
H‐89 reverses the protective effect of MRS1523 against WMI and cognitive impairment after BCAS. (A) Grouping of mice and procedure. (B) NOR test (*n* = 12). (C) Y maze test (*n* = 12). (*D*) Cue‐related FC test (*n* = 12). (E) Context‐related FC test (*n* = 12). (F, G) Representative FA images and quantitative analysis (*n* = 12). (H–J) Representative western blot images and quantitative analysis of PLP1 and MAG (*n* = 5). (K–M) Immunofluorescence staining and quantitative analysis of MBP and LFB staining in the CC, EC, and STR (*n* = 5). Scale bar 200 μm. *p < 0.05, **p < 0.01, ***p < 0.001.

We went on to analyze whether the protective effect of MRS1523 against WMI and cognitive impairment on BCAS could be reversed by H‐89 (Figure 6A). The results showed that the H‐89 treatment abolishes behavioral improvements in BCAS mice induced by MRS treatment in the Y‐maze test, NOR test, and FC test (*p* < 0.01; Figure 6B–E). The MRI DTI examination also showed a reduced FA value in the CC and EC after H‐89 treatment (*p* < 0.01; Figure 6F,G). Meanwhile, compared with MRS1523‐treated BCAS mice, H‐89 treatment decreased the expression of PLP1 and MAG in the CC (*p* < 0.01; Figure 6H–J), and decreased MBP and LFB intensities in CC, EC, and STR (*p* < 0.01; Figure 6K–M). Additionally, we assessed the impact of using H‐89 alone on cognitive behavior and WMI in BCAS mice. The results indicated that H‐89 treatment further exacerbated cognitive impairment and WMI in BCAS mice (*p* < 0.05; Figure S4).

### 3.7 ADORA3 antagonist promotes phagocytosis of myelin debris by primary microglia

**FIGURE 7:**
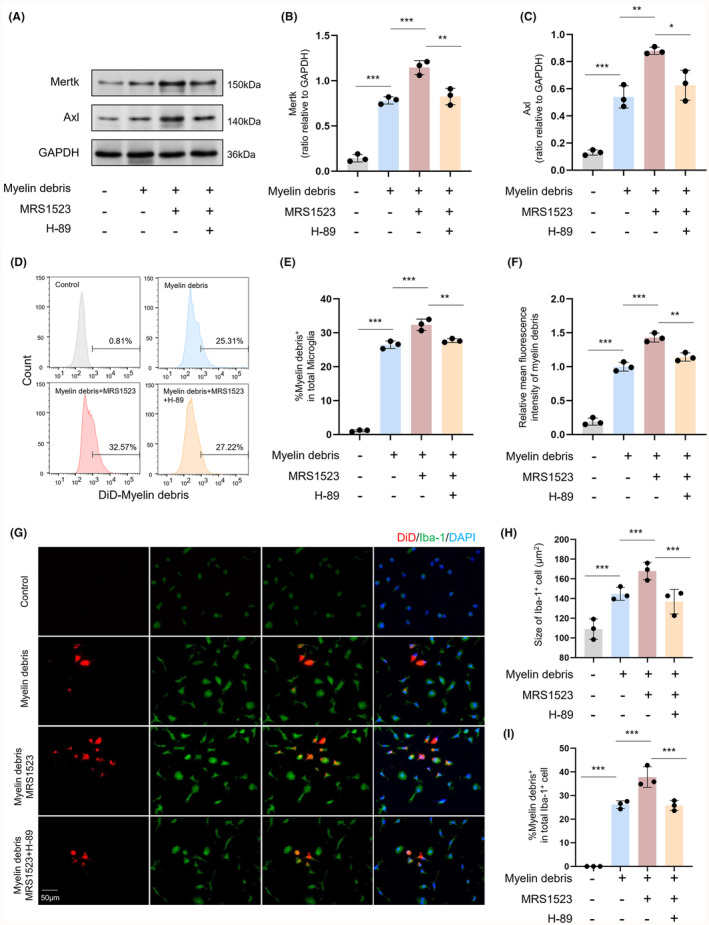
ADORA3 antagonist promotes phagocytosis to myelin debris in primary microglia. (A–C) Representative western blot images and quantitative analysis of Mertk and Axl in primary microglia (*n* = 3). (D, E) Percentage of total microglia that phagocytose myelin debris as detected by FACS (*n* = 3). (F) Relative mean fluorescence intensity of myelin debris in microglia (*n* = 3). (G–I) Immunofluorescence staining and quantification of DiD^+^Iba‐1^+^ cells (*n* = 3). Scale bar 50 μm.*p < 0.05, **p < 0.01, ***p < 0.001.

We further investigated the effects of ADORA3 antagonist on phagocytic function in primary microglia. We detected a significant increase in Mertk and Axl protein expression in primary microglia after the addition of myelin debris (*p* < 0.001). Moreover, MRS1523 treatment increased Mertk and Axl protein expression in microglia after the addition of myelin debris (*p* < 0.01), and H‐29 treatment reduced the pro‐expression effect of MRS1523 Mertk and Axl protein expression in microglia (*p* < 0.05; Figure 7A–C). Further evaluation of the phagocytic ability of microglia toward myelin debris was conducted using flow cytometry and immunofluorescence staining. Consistently, these results demonstrated that MRS1523 treatment enhanced the ability of microglia to phagocytose myelin debris, while H‐29 treatment attenuated this effect of MRS1523 on microglia (*p* < 0.01; Figure 7D–I). Additionally, we assessed the impact of ADORA3 agonist (IB‐MECA) on the phagocytic ability of microglia. Our observations indicated that IB‐MECA treatment reduced the Mertk and Axl protein expression in microglia after myelin debris stimulation (*p* < 0.001; Figure S5A–C). Similarly, flow cytometry and immunofluorescence results visually demonstrated that IB‐MECA decreased the phagocytic ability of microglia toward myelin debris (*p* < 0.001; Figure S5D–I). Furthermore, we investigated whether MRS1523 directly confers protection to myelin. To explore this, an in vitro model of chronic hypoxia in oligodendrocytes induced by CoCl_2_ was established, and immunofluorescence staining for dMBP was performed. The results showed that MRS1523 treatment did not reduce the expression of dMBP in oligodendrocytes after chronic hypoxia (*p* > 0.05; Figure S6). This suggests that the protective effect of MRS1523 on myelin may not be achieved through direct action on oligodendrocytes.

## 4 DISCUSSION

In this study, our analysis revealed that the mRNA expression of ADORA3 was significantly elevated in VaD brain tissue in the GSE122063 dataset and ADORA3 was identified as a key gene for VaD. We revealed that ADORA3 antagonist can ameliorate WMI and cognitive impairment after BCAS by improving Mertk‐mediated microglial phagocytosis to myelin debris, and Mertk‐mediated microglial phagocytosis is promoted by the activation of cAMP/PKA/p‐CREB pathway.

With the advancement of microarray and bioinformatics technologies, an increasing number of bioinformatics analysis methods are employed to explore the potential mechanisms and biomarkers of neurological diseases.^34^, ^35^ Following the identification of ADORA3 through protein–protein interaction analysis and the application of the clustering coefficient and eccentricity algorithms, our focus shifted to investigating the alterations in adenosine and ADORA3 following cerebral ischemia and hypoxia. Adenosine, an endogenous purine nucleoside, is released in substantial quantities during hypoxic or ischemic conditions.^14^ Adenosine acts by activating all subtypes of the receptor and exerting their respective protective or deleterious effects after selective activation or inhibition.^13^, ^36^, ^37^ Early study in gerbils injected with ADORA3 agonists after stroke showed impairment of postischemic blood flow, extensive hippocampal neuronal destruction, and increased mortality,^38^ suggesting that the activation of ADORA3 played a harmful role in the process of acute ischemic injury. Furthermore, in an in vitro model simulating cerebral ischemia through hypoxia and glucose deprivation (OGD), ADORA3 antagonists prevented injury‐induced synaptic failure and excitotoxic damage.^39^, ^40^, ^41^ These studies suggest a possible protective role of ADORA3 antagonist after ischemia. On this basis, we explored the role of ADORA3 antagonist in VaD and demonstrated that ADORA3 antagonist also has therapeutic effects on chronic ischemia‐induced cognitive impairment and WMI.

It is critical to understand the expression of ADORA3 in neural cells for studying the function of ADORA3. Singh AK and colleagues showed that ADORA3 is expressed in all types of neuronal cells in mouse cortical tissues using RNA scope and found elevated expression of ADORA3 in mouse microglia, astrocytes, and oligodendrocytes after cisplatin treatment,^42^ suggesting that ADORA3 may mainly affect function of glial cells. Since chronic ischemia‐induced WMI is an important pathological change in VaD, ADORA3 expression in microglia, oligodendrocytes, and astrocytes in white matter is concerned. Our results revealed that ADORA3 exhibited the highest fidelity in microglia within the white matter, as indicated by Kevin W's database. Moreover, ADORA3 expression was notably elevated in microglia within the white matter in mice after BCAS, indicating that ADORA3 likely modulates the function of microglia in chronic ischemia‐induced WMI.

The Mertk and Axl belong to the TAM (Tyro3, Axl, and Mertk) family and are specialized phagocytic cell receptors expressed on the surface of microglia and other immune cells.^43^ All phagocytic processes begin with the exposure of “eat me” signals from apoptotic cells or fragments. For instance, phosphatidylserine exposed to apoptotic cell/myelin debris can serve as a potent “eat me” signal, triggering rapid recognition and clearance by microglia through Mertk.^44^, ^45^ Mertk and Axl play crucial roles in efficiently clearing apoptotic cells and debris, which is essential for promoting neurogenesis and repair.^46^ Moreover, the phagocytosis of myelin and subsequent remyelination in multiple sclerosis (MS) also requires Mertk, and the functional role of Mertk in WMI has been repeatedly confirmed in MS.^47^, ^48^ After chronic cerebral ischemia, the number and phagocytosis capacity of microglia increase compensatorily in response to the generated myelin debris,^5^, ^7^ which was also observed in our study that BCAS mice with enhanced microglial phagocytosis.

Enhanced phagocytosis of myelin debris by microglia can alleviate the damage caused by myelin debris and is an important treatment for VaD.^5^, ^49^ Our previous studies have also reported that upregulation of Mertk expression enhances the phagocytic function of microglia, promoting the engulfment of myelin debris, thus reversing pathological changes in white matter and exerting cognitive protective effects.^5^ Therefore, it is evident that the Mertk–Axl pathway is a key pathway involved in regulating the phagocytic function of microglia in VaD. Meanwhile, it has been proved that microglial phagocytosis in mouse brains is promoted by cAMP‐PKA‐dependent phosphorylation.^15^, ^16^, ^17^ It has also been reported in phagocytic retinal pigment epithelium that activation of the Mertk–Axl pathway is mediated by cAMP‐PKA‐dependent CREB phosphorylation.^50^ In our study, we not only found that ADORA3 antagonist could promote microglia phagocytosis by upregulating the expression of Mertk and Axl but also confirmed that Mertk‐mediated phagocytosis of microglia was modulated through cAMP/PKA/p‐CREB. Additionally, H‐89, functioning as a cAMP/PKA pathway inhibitor, has the capacity to reverse the protective effect of the ADORA3 antagonist on WMI in VaD by attenuating the phagocytic function of microglia. Evidence from peripheral Schwann cells suggests that H‐89 abolishes the drug‐induced promotion of remyelination by blocking the cAMP/PKA pathway,^51^ which further supports our conclusion. Furthermore, our observations revealed that H‐89 exacerbated cognitive impairment and WMI in BCAS mice, reaffirming the pivotal role of the cAMP/PKA pathway in the treatment of WMI. Nevertheless, it is plausible that H‐89 may exert additional effects that remain undiscovered, necessitating further exploration.

It is worth noting that the phagocytic activity of microglia exhibits duality, as excessive enhancement of phagocytic function may cause damage to myelin sheaths. Zhang et al.^52^ demonstrated in a rat model of VaD induced by bilateral common carotid artery occlusion that enhanced pro‐inflammatory and phagocytic functions of microglia (evidenced by increased CD86 and CD68 expression) were detected at 7 and 14 days, accompanied by aggravated myelin damage. Upon blockade of the C3‐C3aR pathway, they observed attenuation of neuroinflammation and reduction in myelin damage. We speculate that the damage to the myelin observed here may be attributed to the combined pro‐inflammatory and phagocytic functions of microglia in the early stages of VaD, with inflammation likely exerting a more dominant influence. However, our observations primarily focus on the phagocytic activity of microglia 2 months after BCAS, and we have validated that enhancing microglial phagocytosis can ameliorate WMI in BCAS mice. These findings also prompt us to consider the temporal dynamics of microglial phagocytic activity. Early phagocytosis may contribute to damage, while later phagocytosis predominantly plays a protective role. Importantly, this suggests that the timing of drug administration should be carefully considered in therapies aimed at improving WMI by enhancing microglial phagocytosis.

In primary microglia, we have confirmed that ADORA3 antagonist can enhance the phagocytic activity of microglia by upregulating the expression of Mertk and Axl. Additionally, we conducted further investigations into the effects of ADORA3 agonists (IB‐MECA) on microglial phagocytic function. Our findings suggest that ADORA3 agonists can decrease the expression of Mertk and Axl in microglia, thereby inhibiting the phagocytosis of myelin debris by microglia. These results support the notion that adenosine may act as a negative regulator of microglial phagocytosis when binding to ADORA3. Furthermore, as ADORA3 expression increases in oligodendrocytes in mice after BCAS, we conducted an analysis of the direct effects of ADORA3 inhibitors on oligodendrocytes. We found that treatment with MRS1523 did not alter the expression of dMBP in oligodendrocytes after chronic hypoxia model, suggesting that ADORA3 antagonist may not directly affect oligodendrocytes and hinting that its protective effects on myelin are not achieved through direct action on oligodendrocytes. These results further support the core conclusion that ADORA3 antagonist exerts its protective effects by enhancing microglial phagocytosis of myelin debris. We speculate that the increased expression of ADORA3 in oligodendrocytes after BCAS may be related to compensatory proliferation and differentiation of OPCs, which warrants further investigation in the future. Moreover, understanding the role of ADORA3 genes in neurons in VaD will be crucial for further exploration of the application of ADORA3‐related drugs in translational research.

The present study also has some limitations: first, the BCAS model mimics VaD only from the perspective of chronic ischemic WMI and does not fully reflect the pathological changes in VaD patients. Second, interspecies variability in ADORA3 exists,^53^ and the findings of ADORA3 antagonists in mice require further validation in clinical patients. In addition, BCAS lacks a well‐matched vitro model, and our use of myelin debris stimulation to mimic the in vivo demyelination process does not adequately reflect the complex pathogenesis that is observed in vivo. The role of ADORA3 antagonist in VaD needs to be further explored.

## 5 CONCLUSION

In conclusion, our study confirms the important role of ADORA3 in the pathogenesis of VaD and reveals the mechanisms underlying the white matter and cognitive protective effects of ADORA3 antagonists in BCAS mice, which provides potential therapeutic agents and new research directions for the future treatment of chronic cerebral ischemia and VaD.

## AUTHOR CONTRIBUTIONS

Yun Xu and Yuhao Xu designed this study; Yuhao Xu wrote the draft manuscript; Yuhao Xu, Limoran Tang, Liang Sun, Yujie Hu, Shengnan Xia, Xinyu Bao, and Haiyan Yang performed the experiments; Zhi Zhang and Liang Sun provided help with data analyses. Yun Xu and Chao Zhou critically reviewed and offered insights for this manuscript. All authors have thoroughly examined and endorsed the final version of the manuscript.

## FUNDING INFORMATION

This research was supported by the National Natural Science Foundation of China (81920108017, 82130036, and 82101431), the STI2030‐Major Projects (2022ZD0211800), Jiangsu Province Key Medical Discipline (ZDXK202216), and the Key Research and Development Program of Jiangsu Province of China (BE2020620).

## CONFLICT OF INTEREST STATEMENT

The authors have disclosed that they have no competing interests. Yun Xu serves as an Editorial Board Member of CNS Neuroscience and Therapeutics and is also a co‐author of this manuscript. In order to mitigate potential bias, Yun Xu was not involved in any editorial decision‐making processes related to the acceptance of this article for publication.

## Supporting information


Supporting Information S1.

## Data Availability

The data that support the findings of this study are available from the corresponding author upon reasonable request.
