# Correction to “Inhibition of ADORA3 Promotes Microglial Phagocytosis and Alleviates Chronic Ischemic White Matter Injury”

**DOI:** 10.1111/cns.70326

**Published:** 2025-04-16

Xu Y, Tang L, Zhou C, et al. “Inhibition of ADORA3 promotes microglial phagocytosis and alleviates chronic ischemic white matter injury”. *CNS Neuroscience & Therapeutics*. 2024;30(5): e14742. https://doi.org/10.1111/cns.14742.

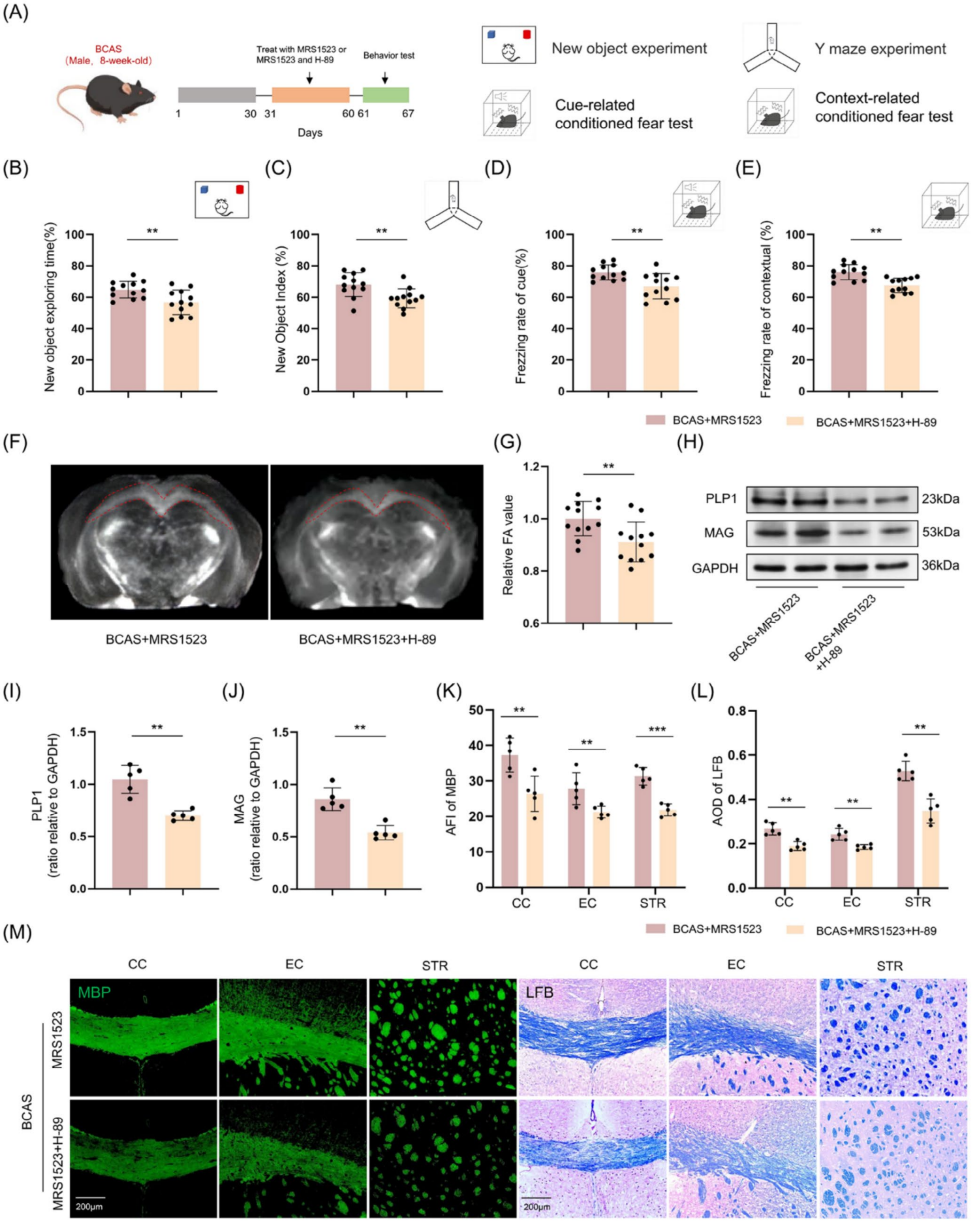

In Figure 6, the LFB‐stained image in panel M was inadvertently replaced with an incorrect version during figure assembly; the correct version has been provided and does not affect the experimental findings.

We apologize for this error.

