# Supplementary material for: Inhibition of ADORA3 promotes microglial phagocytosis and alleviates chronic ischemic white matter injury
Source: CNS Neurosci Ther. 2024 May 7;30(5):e14742. doi: 10.1111/cns.14742 (PMC11076989; doi:10.1111/cns.14742)

Full unedited blot

1. Full unedited blot for Figure 2C

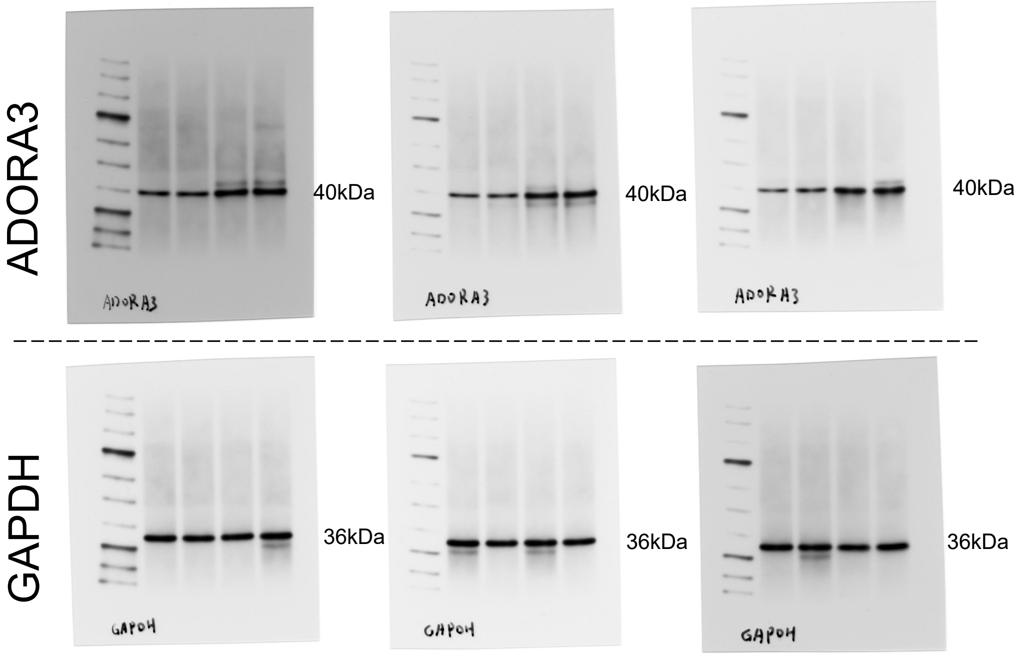

2. Full unedited blot for Figure 3H

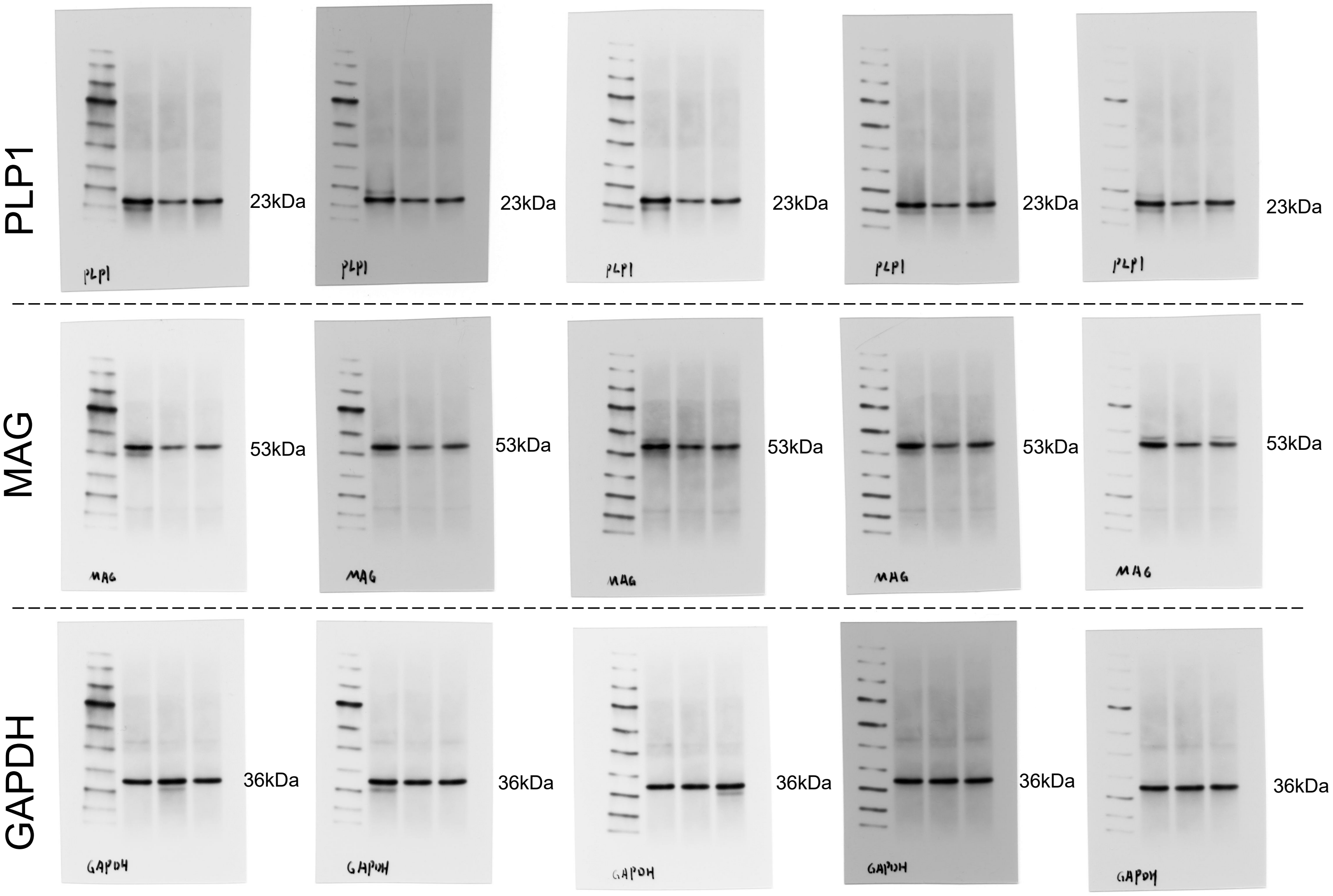

3. Full unedited blot for Figure 4A

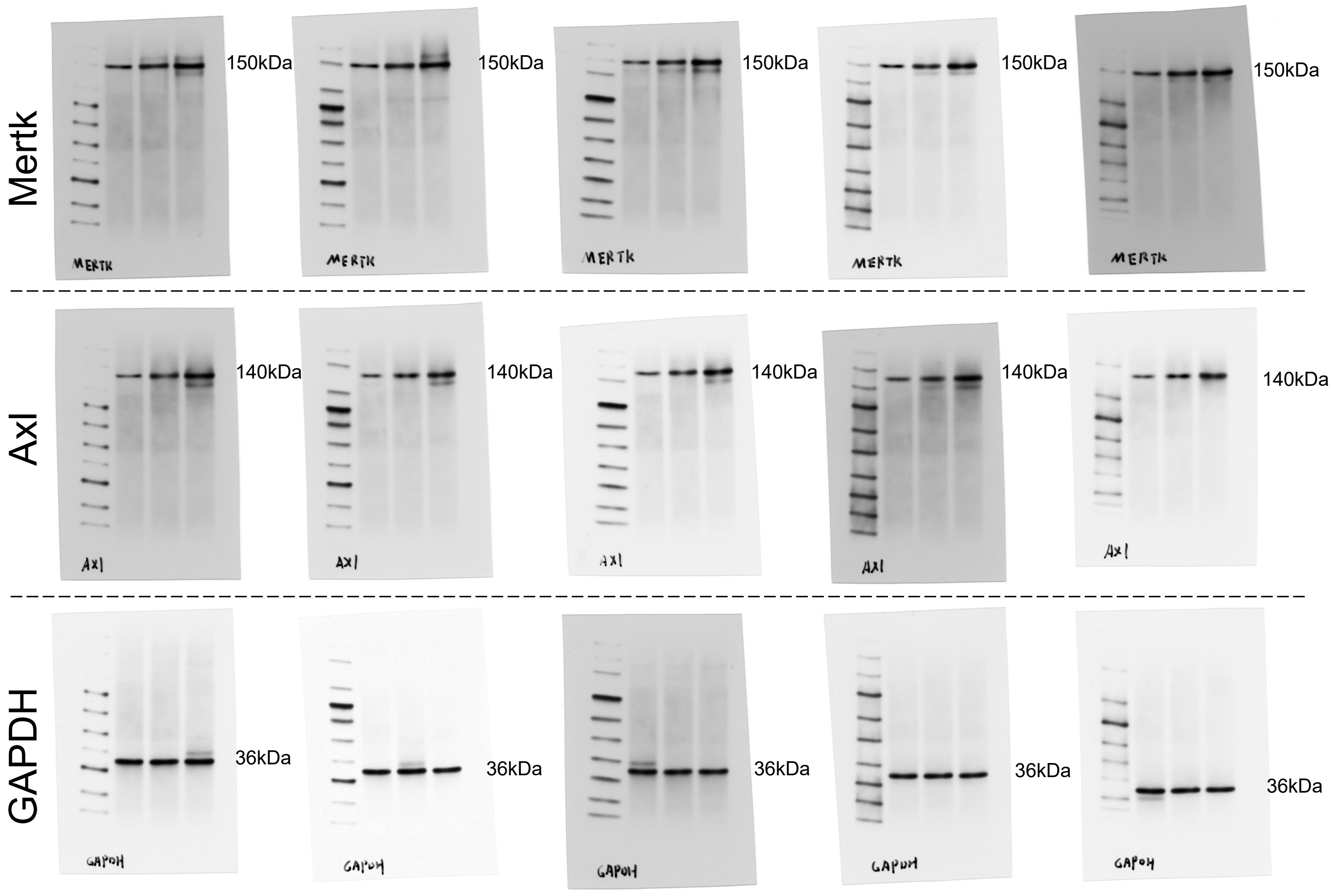

4. Full unedited blot for Figure 5C

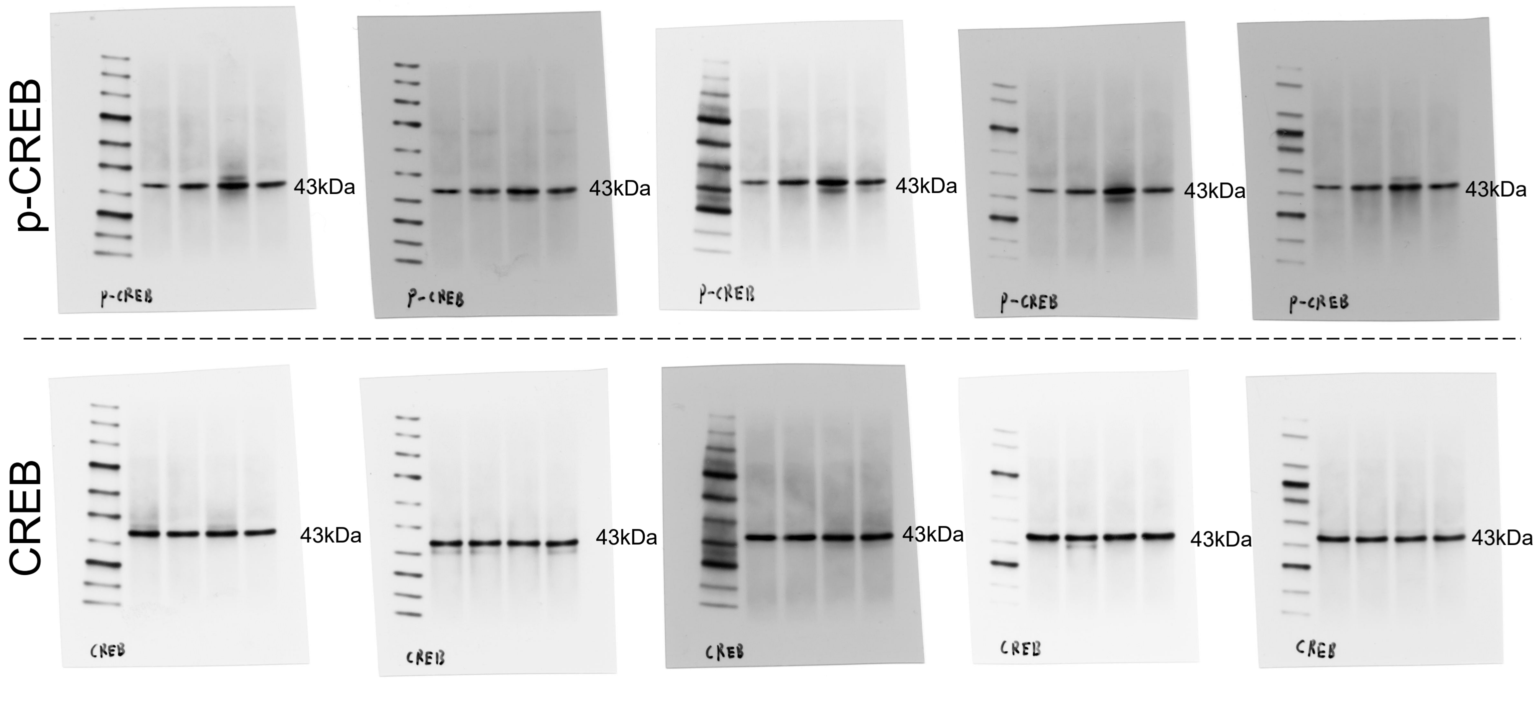

4. Full unedited blot for Figure 5C

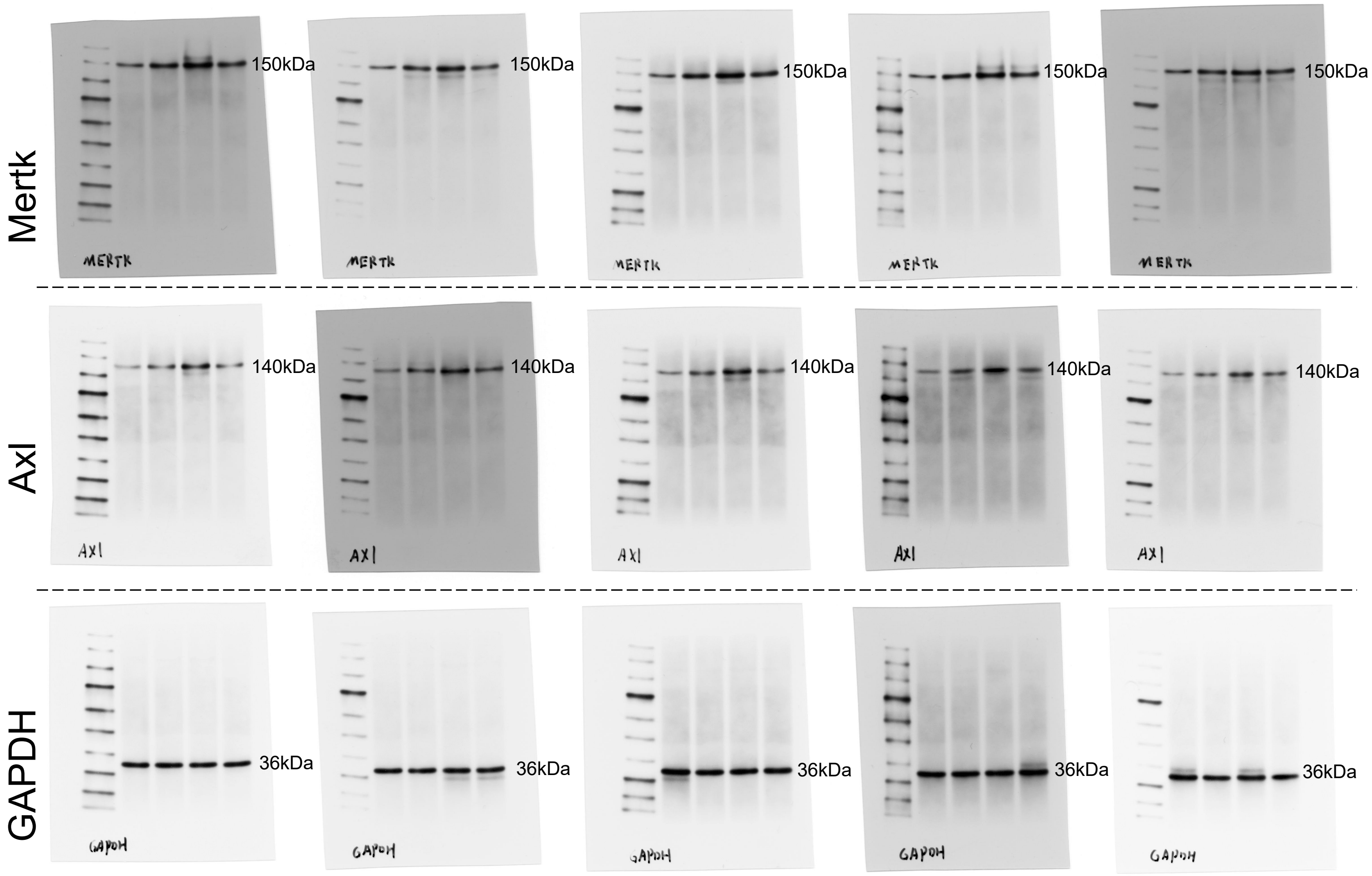

5. Full unedited blot for Figure 6H

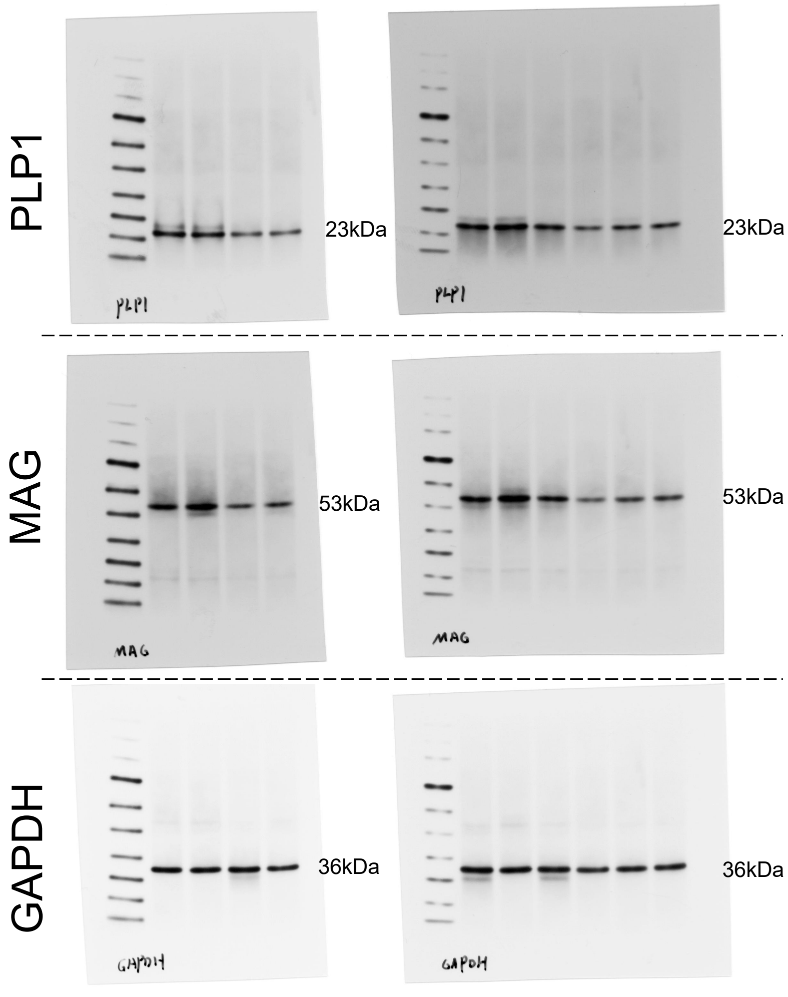

6. Full unedited blot for Figure 7A

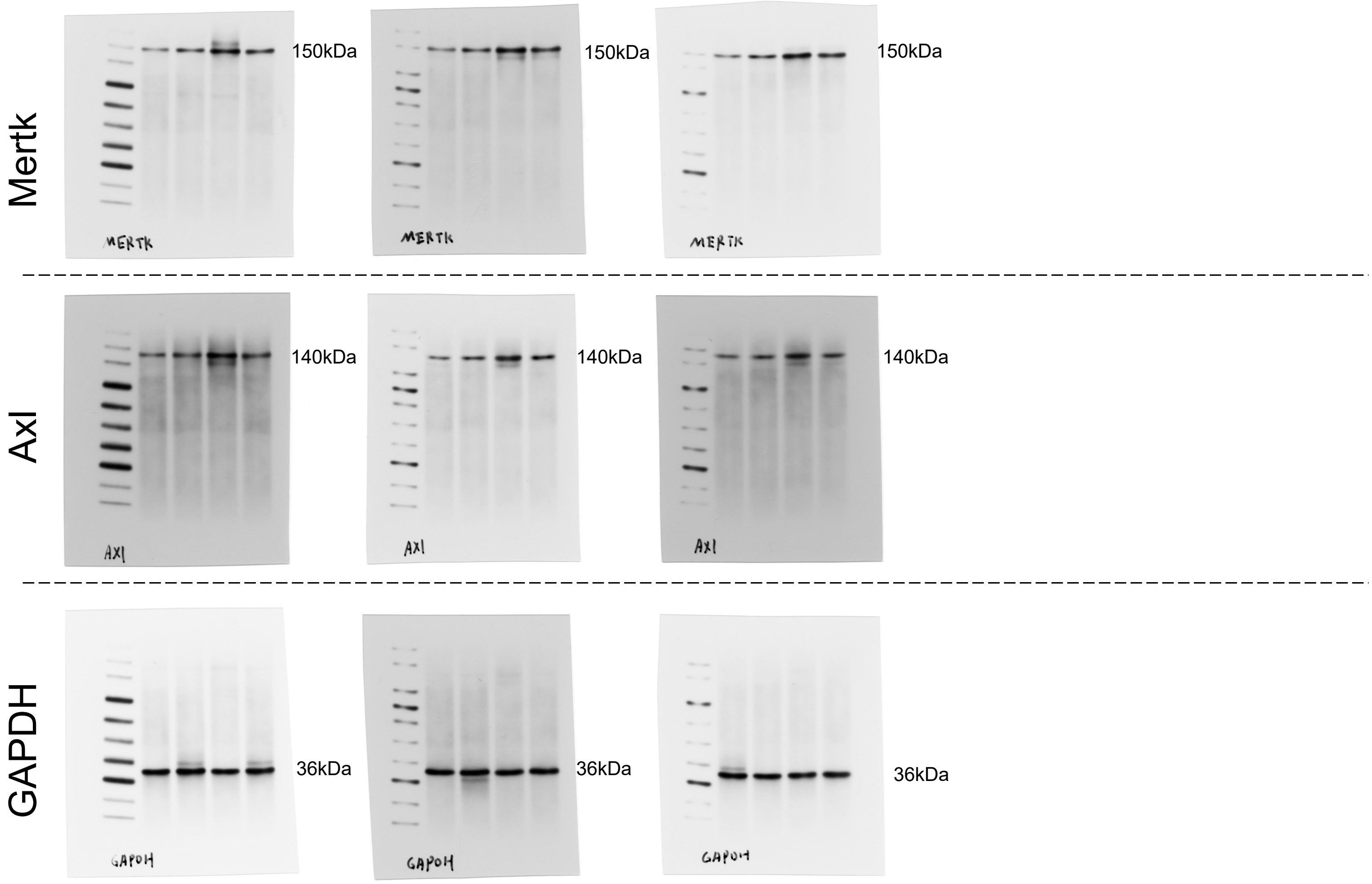

7. Full unedited blot for Figure S5A

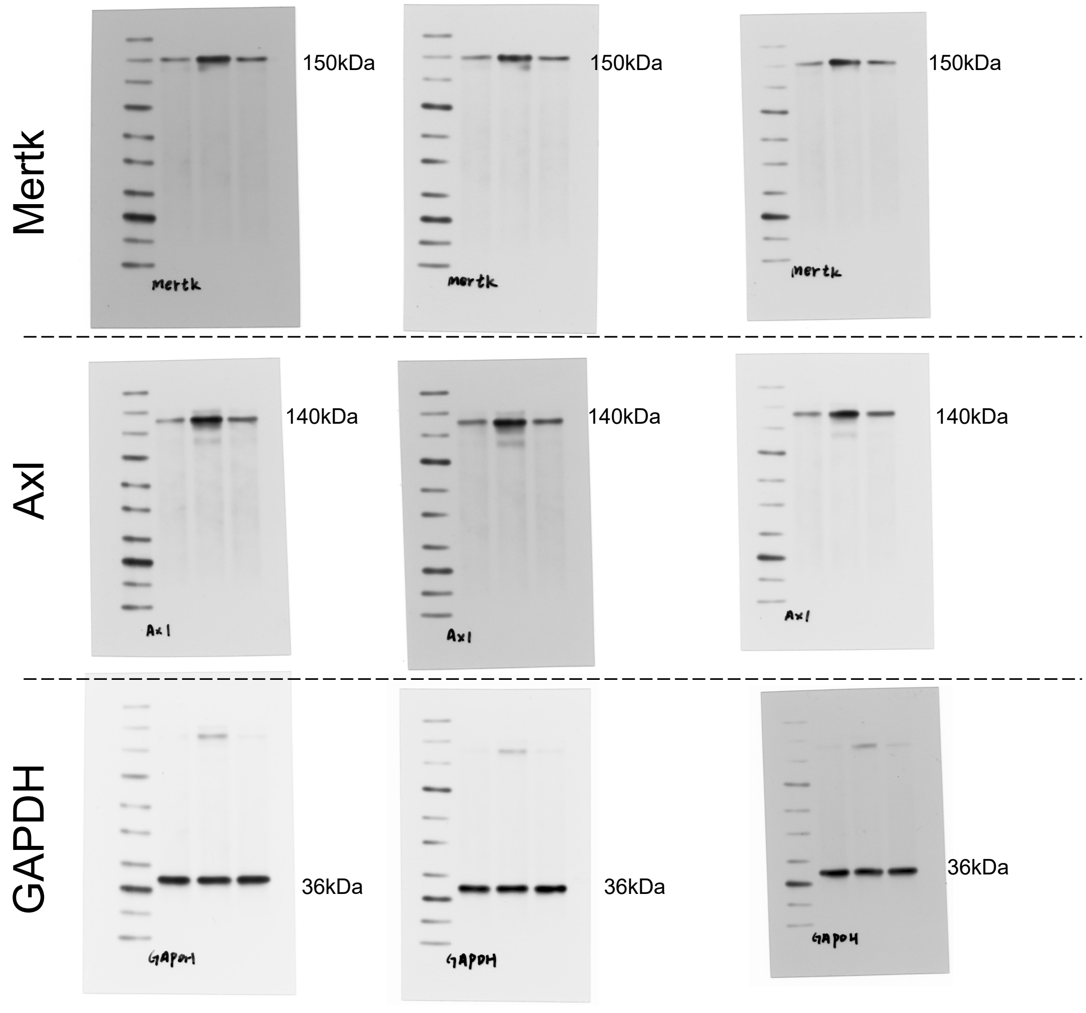

Supplement: Supplementary file 1 — Supporting Information S1. [file CNS-30-e14742-s001.zip › Supplemental Files-bolt.pdf]
