# Supplementary material for: Inhibition of ADORA3 promotes microglial phagocytosis and alleviates chronic ischemic white matter injury
Source: CNS Neurosci Ther. 2024 May 7;30(5):e14742. doi: 10.1111/cns.14742 (PMC11076989; doi:10.1111/cns.14742)
Supplement: Supplementary file 1 — Supporting Information S1. [file CNS-30-e14742-s001.zip › Supplemental Files-FIGURE S1-6.doc]

**Supplemental FIGURE S1-6**

**FIGURE S1**

**FIGURE S1** Representative images of laser speckle contrast imaging.

**FIGURE S2**

**FIGURE S2** The precise locations for image capture in MBP immunofluorescence staining and LFB staining include the CC (Corpus Callosum), EC (External Capsule), and STR (Striatum) regions.

**FIGURE S3**

**FIGURE S3** MRS1523 treatment has no effect on body weight, cognition, or myelin in Sham mice. (A) NOR test (n=10). (B) Y maze test (n=10). (C) Cue-related FC test (n=10). (D) Context-related FC test (n=10). (E-G) Immunofluorescence staining and quantitative analysis of MBP and LFB staining in the CC, EC and STR (n=5). Scale bar 200μm. "ns" indicates no significance (*p*>0.05).

**FIGURE S4**

**FIGURE S4** H-89 exacerbates WMI and cognitive impairment in BCAS mice. (A) NOR test (n=10). (B) Y maze test (n=10). (C) Cue-related FC test (n=10). (D) Context-related FC test (n=10). (E-G) Immunofluorescence staining and quantitative analysis of MBP and LFB staining in the CC, EC and STR (n=5). Scale bar 200μm. **p*<0.05, ***p*<0.01, ****p*<0.001.

**FIGURE S5**

**FIGURE S5** ADORA3 agonist (IB-MECA) inhibits phagocytosis to myelin debris in primary microglia. (A-C) Representative western blot images and quantitative analysis of Mertk, Axl in primary microglia (n=3). (D-E) Percentage of total microglia that phagocytose myelin debris as detected by FACS (n=3). (F) Relative mean fluorescence intensity of myelin debris in microglia (n=3). (G-I) Immunofluorescence staining and quantification of DiD^+^Iba-1^+^ cells (n=3). Scale bar 50μm. **p*<0.05, ***p*<0.01, ****p*<0.001.

**FIGURE S6**

**FIGURE S6** MRS1523 treatment did not reduce dMBP expression in oligodendrocytes after chronic hypoxia induced by Cocl_2_. (A-B) Immunofluorescence staining and quantitative analysis of dMBP intensity (n=3). Scale bar 20μm. "ns" indicates no significance (*p*>0.05).
